# CHANGE IN SOCIAL INTERACTION AND MENTAL HEALTH AMONG OLDER AMERICANS DURING COVID-19 PANDEMIC

**DOI:** 10.1093/geroni/igac059.1891

**Published:** 2022-12-20

**Authors:** Krystyna Keller, Jielu Lin, Melissa Zajdel, Fiona Gilpin Macfoy, Philip Shaw, Brenda Curtis, Lyle Ungar, Laura Koehly

**Affiliations:** National Institutes of Health, Bethesda, Maryland, United States; National Institutes of Health, Bethesda, Maryland, United States; National Institutes of Health, Bethesda, Maryland, United States; National Institutes of Health, Bethesda, Maryland, United States; National Institutes of Health, Bethesda, Maryland, United States; National Institutes of Health, Baltimore, Maryland, United States; University of Pennsylvania, Philadelphia, Pennsylvania, United States; National Institutes of Health, Bethesda, Maryland, United States

## Abstract

Recent research has shown the mental health consequence of social distancing during the COVID-19 pandemic, but longitudinal data are relatively scarce. It is unclear whether the pattern of isolation and elevated stress seen at the beginning of the pandemic persists over time. This study evaluates change in social interaction over six months and its mental health impact among older adults. We drew data from a panel study with six repeated assessments of social interaction and mental health conducted monthly May through October 2020. The sample included a total of 380 White, Black and Hispanic participants aged 50 and over, of whom 33% had low income, who residing in fourteen U.S. states with active stay-at-home orders in May 2020. The analysis examined how change in living arrangement, in-person interaction outside the household, quality of relationship with family and friends, and perceived social support affected trajectories of isolation stress, COVID worry and sadness. While their living arrangements and relationship quality remained stable, older adults experienced fluctuations in perceived social support and increases in in-person conversations outside the household. Living with a spouse/partner stabilized isolation stress and COVID worry over time. Individuals with better relationship quality with friends became happier over time. Changes in social support were associated with greater fluctuations in isolation stress and COVID worry. During the pandemic, social interactions are protective and lack of stability in feeling supported makes older adults vulnerable to stress. Efforts should focus on (re)building and maintaining companionship and support to mitigate the pandemic’s negative impact.

